# Is There a Social Prejudice against Dentists?

**Published:** 1904-10-15

**Authors:** 


### IS THERE A SOCIAL PREJUDICE AGAINST DENTISTS?

In one of the fashionable clubs of New York City an incident recently occurred that illustrates one of the peculiarities of modern social life. Although no hint of it was permitted to reach the outside world at the time, the club was kept in a turmoil for several weeks by the proposal of a certain man for membership. Only one objection was raised against him, and that had nothing to do with his personality. His bitterest opponents freely conceded that in so far as education and character could make a gentleman he was one, and moreover, it was generally recognized that he had the wealth necessary to maintain the standards set by the club. Nevertheless the sentiment against his admittance to membership was overwhelming, and his application was withdrawn. It was his profession, and his profession solely, that caused all the trouble. The applicant was a dentist.

In discussing the incident one of the members of the club who had been most active in opposing the dentist said: "I am free to confess that my opposition was based upon blind prejudice, but in justice to myself I think I may say my prejudice is merely the reflection of the prejudice of my associates. By nature I do not think I am a snob. At the same time I know none of my friends would care to meet a dentist socially. I should never dream of inviting a dentist to my home, and one does not care to have as one's fellow club member a man one is not free to take everywhere and invite everywhere. Look at the facts. Can you point to a dentist anywhere in the United States or England who occupies any sort of a social position? I grant that the social prejudice against dentists is unreasonable. Modern dental surgery is undoubtably a science the mastery of which requires a high order of intelligence, and it is difficult to see why a man who practices it should not rank with the ordinary surgeon and physician. Yet the ordinary surgeon and physician are gladly received where the door is slammed in the face of the dentist. I notice that a Chinaman of noble birth who has just written a book about the United States was struck by this peculiarity of our social life. He inquired diligently as to its reason, and the only explanation it seems that he ever got was that it was part of a dentists' duties to clean teeth, it being argued that no gentleman of culture and refinement would ever stoop to such work. The argument appears not to have impressed the Chinaman, and I don't suppose it will impress anybody else who reasons. A physician is frequently called upon to clean a patient's stomach, and friends of the dentist may well say that sort of work has far more objectionable features than the cleaning of teeth."

This is undoubtedly an extreme case, but there is enough significance in the incident and enough truth in the criticisms to furnish the dental profession food for serious thought. It is all very well to talk of dentistry being a learned profession, but we will never make it such nor raise it to the position it should occupy in the minds of the community so long as we continue in this self-satisfied state and blind our eyes to the true condition of affairs. Even the most ardent champions of dentistry must admit that its members do not rank with those of other professions nor even with the average men in other walks of life. We do not believe the low estimate which the public puts upon dentists is due to the nature of their work, but rather to their lack of education and culture. Forty years ago Dr. L. D. Shepard said, "Seriously, as a profession, do we read enough? Are we up to the times as a learned profession in the great throes of thought, science and progress? One grand curse upon our profession from its beginning to the present day has been the lamentable and very general ignorance of its members." Could not the same be said to-day with quite as much truth, and what is the remedy? —Editorial in August *Digest.*

